# Prenatal ultrasound findings and clinical outcomes of uniparental disomy: a retrospective study

**DOI:** 10.1186/s12884-024-06493-0

**Published:** 2024-04-18

**Authors:** Cui-Yi Wu, Yi Zhou, Xia Yin, Ruan Peng, Hong-Ning Xie

**Affiliations:** 1 Department of Ultrasonic Medicine, Fetal Medical Centre, The First Affiliated Hospital of Sun Yat-sen University, Guangzhou, Guangdong China; 2 Department of Obstetrics, The First Affiliated Hospital of Sun Yat-sen University, Guangzhou, Guangdong China

**Keywords:** Chromosome, Fetal abnormalities, Fetal growth restriction, Prenatal ultrasound

## Abstract

**Background:**

Uniparental disomy is the inheritance of a homologous chromosome pair or part of homologous chromosomes from only one parent. However, the clinical significance of uniparental disomy and the difference among the prognosis of involvement of different chromosomes remain unclear.

**Objective:**

To assess the associated prenatal ultrasound presentations and clinical outcomes of uniparental disomy on different chromosomes and to analyze the relationship between prenatal ultrasound markers and clinical outcomes.

**Study design:**

We retrospectively analyzed data from fetuses with uniparental disomy diagnosed using chromosome microarray analysis with the Affymetrix CytoScan HD array at our institution between January 2013 and September 2022. The relationship between prenatal ultrasound findings, the involved chromosome(s), and clinical outcomes was evaluated.

**Results:**

During the study period, 36 fetuses with uniparental disomy were diagnosed, and two cases were excluded for non-available postnatal data. Finally, 34 fetuses were included in our study, of which 30 (88.2%) had uniparental disomy occurring on a single chromosome, while four (11.8%) were identified with uniparental disomy on different chromosomes. The most frequently involved chromosomes were chromosomes 16, X and 2, which presented in 8 (23.5%), 5 (14.7%) and 4 (11.8%), respectively. Prenatal ultrasound abnormalities were detected in 21 fetuses, with the most common category being multiple abnormalities (12 (57.1%)). Fetal growth restriction was identified in 14 (41.2%) fetuses, all of which coexisted with other abnormal findings. The rate of adverse perinatal outcomes in patients with uniparental disomy and fetal abnormalities was significantly higher than those without abnormalities (76.2% versus 15.4%, *P* = 0.002). The incidence of fetal or neonatal death was significantly higher in fetuses with fetal growth restriction than those without (85.7% versus 30.0%, *P* = 0.004).

**Conclusions:**

The prognosis of fetuses with uniparental disomy combined with fetal abnormalities, especially fetal growth restriction, was much poorer than those without.

**Supplementary Information:**

The online version contains supplementary material available at 10.1186/s12884-024-06493-0.

**Why was this study conducted?**

The clinical significance of uniparental disomy is unclear. We assessed the prenatal ultrasound presentations and clinical outcomes of uniparental disomy and analyzed the relationship between them.

**What are the key findings?**

We found that the prognosis of fetuses with uniparental disomy combined with fetal abnormalities, especially fetal growth restriction, was much poorer than that of fetuses without. Sonographic presentations should be combined with the results of invasive prenatal diagnoses to provide useful information for clinical counseling.

## Introduction

Uniparental disomy (UPD) is defined as the inheritance of a pair or part of chromosomes from only one parent and was first proposed in 1980 [1]. The first molecularly proven case was published in 1987 by Créau-Goldberg [2]. According to the origin of the chromosomes, UPD cases can be divided into maternal UPD (mat UPD) and paternal UPD (pat UPD), with mat UPD being approximately twice as common as pat UPD [3]. The mechanisms may be trisomy rescue, monosomy rescue, gamete complementation, or other rare mechanisms [4, 5]. The occurrence of UPD in the population is relatively common, at a rate of approximately 1/2000 [6]. The clinical phenotype of UPD, caused by changes in gene expression, includes imprinted genetic disorders, mutations in harmful autosomal recessive disorders, residual aneuploidy, or mosaic trisomy [7]. Previous studies have reported that UPD causes different clinical phenotypes according to chromosomal involvement. When UPD occurs on chromosomes 6, 7, 11, 14, 15, or 20, it causes imprinted genetic disorders such as pat UPD 6 (transient neonatal diabetes mellitus), mat UPD 7 (Silver-Russell syndrome), and pat UPD 11p (Beckwith-Wiedemann syndrome) [8]. However, the pathogenic phenotype is not always identified in all UPD cases; thus, the clinical significance of UPD and the difference among the prognosis of involvement of different chromosomes remain ambiguous [9]. 

Currently, UPD can be detected using single-nucleotide polymorphism (SNP)-based chromosome microarray analysis (CMA) technology, microsatellite analysis, and trio exome sequencing. The advantage of SNP arrays is that they can identify all UPDs across all chromosomes, including segmental UPDs in the case of isodimorphism [10, 11]. CMA encompasses microarray-comparative genomic hybridization (CGH) analysis and SNP arrays and is widely recommended as the first-line prenatal genetic analysis for those with structural abnormalities [12]. In our institution, the indications for prenatal genetic testing usually include abnormal fetal structures or development detected using ultrasound, risk factors for genetic variations (assisted reproductive technology, advanced maternal age, and adverse pregnancy history), and abnormal findings on serological screening and noninvasive prenatal testing (NIPT). The different prenatal ultrasonographic findings may provide clues for predicting different clinical outcomes.

This study aimed to analyze the association between prenatal ultrasonographic findings and clinical outcomes in fetuses with UPD. Additionally, we assessed the relationship between prenatal ultrasound markers, involvement of different chromosomes, and the prognosis of fetuses with UPD to provide essential information for genetic counseling.

## Materials and methods

This retrospective study enrolled all patients diagnosed with UPD using CMA at our institution between 2013 and 2022. Amniotic fluid was obtained by amniocentesis at 16–25^+ 6^ gestational weeks or umbilical cord blood was obtained by cordocentesis at > 26 gestational weeks to perform CMA. Indications for prenatal microarray analysis included fetal abnormalities, history of previous adverse pregnancy, advanced maternal age, parental genetic disease, and abnormal findings on serological screening and NIPT. Informed consent was obtained from all patients before they underwent invasive prenatal diagnosis. Specific post-test counseling was provided. This study was approved by the Ethics Committee of our institution ([2020]060) and was conducted in accordance with the Declaration of Helsinki (as revised in 2013).

Microarray-CGH analysis and SNP array technologies were used in each case, which were performed using an Affymetrix CytoScan HD array (Affymetrix Inc., Santa Clara, CA, USA) with a high resolution of 100 kb. The results were analyzed using the Affymetrix Chromosome Analysis Suite (genome build 37). Copy number variations (CNVs) were compared to those in public databases. Published articles were reviewed when necessary. When the blocks of homozygosity on a single chromosome were larger than the average blocks of homozygosity throughout the whole genome, they were considered UPDs. CNVs larger than 100 kb and regions of homozygosity with a fragment length of over 10 MB were reported by the laboratory. CNVs were categorized as pathogenic, benign, or unknown clinical significance.

A detailed ultrasonographic evaluation of the fetal anatomy and biological measurements, including echocardiography, were performed in each case. Data including basic information, maternal serum screening results, NIPT results using cell-free fetal DNA in maternal plasma, ultrasonographic presentations, CMA findings, pregnancy outcomes, and postnatal follow-up data were collected by searching the medical record system of our institution. If the patients delivered at our institution, pregnancy outcomes were obtained from the delivery records. Otherwise, the patients were followed up by telephone. Unfavorable pregnancy outcomes included termination of pregnancy, selective reduction of twin pregnancies, miscarriage, and perinatal death. A favorable pregnancy outcome was children being alive at the time of this writing.

Five stratified statistical analyses were carried out according to (a) the presence of fetal abnormalities with prenatal ultrasound (yes vs. no); (b) the presence of fetal growth restriction (FGR) (yes vs. no); (c) the presence of multiple or isolated fetal abnormalities with prenatal ultrasound; (d) involvement of chromosome 16 or 2 vs. other chromosomes involved; (e) the gestational weeks of ultrasound abnormalities detected (at<28 weeks vs. at ≥ 28 weeks).

Statistical analyses were performed using SPSS software (SPSS Inc., Chicago, IL, USA, version 25). Continuous variables are expressed as mean ± standard deviation or median (range) and were analyzed using Wilcoxon rank sum test. Categorical variables are reported as percentages and were analyzed using chi-square analysis or Fisher’s exact test. A univariate logistic regression analysis was performed to analyze the relationship between the risk factors and unfavorable pregnancy outcomes in patients with UPD. Statistical significance was set at *p* < 0.05.

## Results

During the study period, 36 fetuses with UPDs were identified using microarray analysis, and two cases were excluded owing to loss to follow-up. The basic characteristics of the 34 fetuses with UPD in our cohort are shown in Table 1. The median maternal age was 33 (range, 19–44) years. The median gestational age in which fetuses were detected with ultrasound abnormalities was 25^+ 5^ (range, 16–31^+ 5^) weeks, and the statistical significance was not significant between these two groups *(P* = 0.968). The overall rate of assistant reproduction treatment (ART) in these 34 patients was 29.4%, whereas it was 16.7% in the group with unfavorable pregnancy outcomes. Twenty-one (61.8%) patients underwent CMA for fetal abnormalities on prenatal ultrasound, and the remaining 13 (38.2%) patients for other indications. The indications for an invasive prenatal diagnosis in the 34 patients are listed in Table 2. Based on the CMA results of all patients, 30 patients (88.2%) had UPD on a single chromosome, and only four (11.8%) patients had UPDs on different chromosomes: one case was observed on chromosomes 1, 11, 13 and 14, one case was observed on chromosomes 1, 8, 10, 11, 15, 16, 18 and 19, one case was observed on chromosomes 5, 11 and 12 and one case was observed on chromosomes 4, 5 and 16. UPD was observed on chromosomes 16, X, 1, 2, 11, 15, 5, 4, 8, 14, 7,10, 12, 13, 18, 17, and 19, with chromosome 16 being the most frequently involved, followed by chromosome X, chromosome 2, chromosome 1; and their prevalence were 8 (26.7%), 5 (16.7%), 4 (13.3%) and 3 (10.0%), respectively. One patient with multiple UPD fragments on chromosome 2 had mosaic regions of homozygosity, with the proportion of mosaicism ranging from 10 to 30%. Abnormal karyotyping was detected in three cases, including 48,XX,+2mar (inherited from the father); 46,X, inv(Y)(p11.2q12); and 46,XY, inv [9](p11q13). The three patients with abnormal karyotyping all had UPD on a single chromosome.

**Table 1:** Basic characteristics of the cohort

Parameter	Clinical outcome		Total (*N* = 34)	*P*-value
	Unfavorable (*n* = 18)	Favorable (*n* = 16)		
Age (years, Median (Range))	33 (19–44)	31 (27–41)	33 (19–44)	0.646
Gestational weeks* (weeks, Median (Range))	25 (16–31^+ 5^)	27 (17–31^+ 5^)	25^+ 5^ (16–31^+ 5^)	0.968
Previous adverse pregnancies (%)	0	3 (18.8)	3 (8.8)	0.094
Twin pregnancy (%)	4 (22.2)	1 (6.3)	5 (14.7)	0.340
ART (%)	3 (16.7)	7 (43.8)	10 (29.4)	0.134
Abnormal findings of NIPT (%)	4 (22.2)	0	4 (11.8)	0.105
Abnormal findings of serological screening (%)	4 (22.2)	1 (6.3)	5 (14.7)	0.340

SD, standard deviation; NIPT, noninvasive prenatal test; ART, assisted reproductive technology; UPD, uniparental disomy; *P*-value, Wilcoxon rank run test or Fisher’s exact test

*The median gestational age in which fetuses were detected with ultrasound abnormalities

**Table 2:** Indications for invasive prenatal diagnosis in 34 pregnancies with uniparental disomy

Indications for invasive prenatal diagnosis	n (%)
Ultrasound abnormalities	21 (61.8)
Parental genetic disease	3(8.8)
High risk result from maternal serum screening	2 (5.9)
Parental abnormal karyotype	2 (5.9)
Advanced maternal age	2 (5.9)
Previous adverse pregnancies	2 (5.9)
Rh-negative blood	2 (5.9)
Total	34 (100)

The cases were classified based on the most important indications for invasive prenatal diagnosis, and the order of importance is as follows: ultrasound abnormalities, high risk of maternal serum screening, parental abnormal karyotype, parental genetic disease, advanced maternal age, previous adverse pregnancies, and Rh-negative blood

Twenty-one patients (61.8%) presented with abnormalities on prenatal ultrasound screening, the most common being multiple abnormalities (12 (57.1%)), while the others were isolated malformations. Skeletal, cardiovascular, and genitourinary system malformations were each detected in two cases respectively (5.9%). The detailed distributions of ultrasound presentation in each case are shown in Supplement Table 1 (Additional File 1). We found that the prenatal sonographic presentations differed greatly among different involved chromosomes.

In fetuses with abnormalities, 20 (95.2%) UPDs occurred on a single chromosome, mostly involving chromosomes 16 and 2 (7 (33.3%) and 4 (19.0%), respectively), while only one patient had multiple UPDs on different chromosomes. In the group without ultrasound abnormalities, 10 (76.9%) patients had UPD on a single chromosome, whereas three (23.1%) patients had multiple UPDs on different chromosomes. The rate of fetuses that were identified with abnormalities were not significantly different between UPDs involving a single chromosome and UPDs involving multiple chromosomes (*P* = 0.274). Additionally, FGR was detected on prenatal ultrasound in 14 (41.2%) fetuses, all of which were combined with other abnormal ultrasound findings. In cases of UPD with FGR, the most frequently involved chromosomes were 16 and 2 (6 (42.9%) and 4 (28.6%), respectively).

**Fig. 1:**
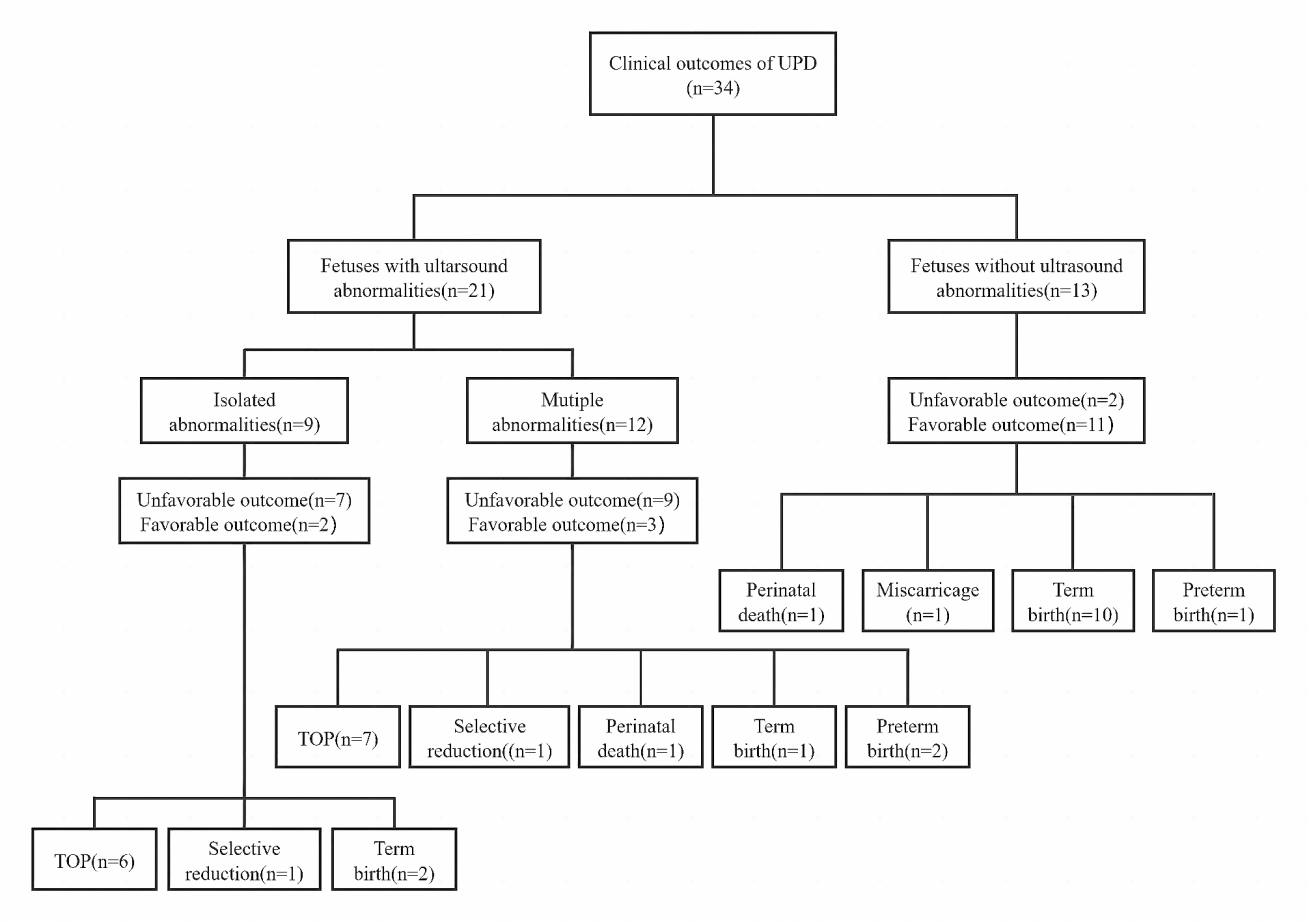
The clinical outcomes of all cases with uniparental disomy in our cohort. TOP, termination of pregnancy; UPD, uniparental disomy Favorable clinical outcomes included term birth and preterm birth; Unfavorable clinical outcomes included termination of pregnancy, selective reduction of twin pregnancies, miscarriage, and perinatal death

The pregnancy outcomes of the 34 fetuses with UPD were as follows: 13 term births, 13 termination of pregnancies, 3 preterm births, 2 perinatal deaths (necrotizing enterocolitis and respiratory distress syndrome), 2 selective reductions of twin pregnancies, and 1 miscarriage due to an accident. The clinical outcomes of all fetuses with UPDs are shown in Fig. 1. Fetuses were divided into the favorable group and the unfavorable group (16 (47.1%) and 18 (52.9%), respectively). In the unfavorable outcome group, the most frequently involved chromosomes were chromosomes 16 and 2 (7 (38.9%) and 4 (22.2%), respectively), whereas chromosome X (4 (25.0%)) was mostly detected in the favorable outcome group.

Two (5.9%) patients had UPD involving multiple chromosomes in the unfavorable outcome group and favorable outcome group respectively, and the rest of patients had UPD involving a single chromosome (16 (47.1%) and 14 (41.2%), respectively). Our study showed no significant difference in the rate of unfavorable outcomes in fetuses with UPDs involving a single chromosome and multiple chromosomes (*P* = 1.000) and no difference in the gestational weeks at which ultrasound abnormalities were detected (*P* = 0.968, Table 1). However, we found that the rate of unfavorable clinical outcomes in fetuses with UPD and ultrasound abnormalities was higher than that in those without ultrasound abnormalities (76.2% versus 15.4%, *P* = 0.002). The incidence of fetal or neonatal death in the fetuses with isolated ultrasound abnormalities was 77.8% (7/9), whereas it was 75.0% (9/12) in the fetuses with multiple ultrasound abnormalities, with no significant difference (*P* = 1.000). Additionally, the incidence of fetal or neonatal death was significantly higher in fetuses with FGR than in those without FGR (85.7% versus 30.0%, *P* = 0.004).

A univariate logistic regression analysis was performed to identify risk factors associated with unfavorable pregnancy outcomes. The results of stratified statistical analyses were showed in Table 3. Univariate logistic regression analysis showed that fetal abnormalities (odds ratio [OR] = 17.60, *P* = 0.002), FGR (OR = 14.00, *P* = 0.004), and chromosome 16 or 2 involvement (OR = 6.810, *P* = 0.017) were associated with unfavorable pregnancy outcomes in UPD.

**Table 3:** Univariate regression analysis of risk factors related to unfavorable pregnancy outcomes of uniparental disomy

Variables	Clinical outcomes		OR (95% CI)	*P*-value
	Unfavorable (*n* = 18)	Favorable (*n* = 16)		
Fetal abnormalities				
Yes	16	5	17.60 (2.88–107.61)	0.002
No	2	11		
Fetal growth restriction				
Yes	12	2	14.00 (2.37–82.72)	0.004
No	6	14		
Multiple abnormalities				
Yes	9	3	4.33 (0.91–20.60)	0.065
No	9	13		
Chromosome involved				
16 or 2	11	3	6.810 (1.41–32.83)	0.017
Others	7	13		
Gestational weeks at ultrasound screening				
<28 weeks	10	14	5.60 (0.97–32.20)	0.063
≥ 28 weeks	8	2		

OR, odds ratio; CI, confidence interval; *P*-value, univariate logistic regression analysis

## Discussion

### Principal findings

This retrospective study analyzed the chromosomal distribution, ultrasonographic features, and clinical outcomes of fetuses prenatally diagnosed with UPD. Most of the UPD cases involved in a single chromosome, including chromosome 16 and X. About half of UPD cases presented with abnormalities on prenatal ultrasound screening. As for the unfavorable outcome group, the most frequently involved chromosomes were chromosomes 16 and 2, which were at the highest risk for coexisting with FGR.

## Results

In our cohort, the median gestational age in which fetuses were detected with ultrasound abnormalities was 25^+ 5^weeks, which is consistent with that in a previous study. The incidence of UPD in spontaneous abortions beyond the 5th gestational week was low [13]. However, microarray analysis was not performed in all cases of spontaneous abortion. The occurrence of UPD should be considered to be a de novo aberration in division of cells with a normal karyotype, therefore, the recurrence risk can even be negligible [14]. Thus, an adverse pregnancy history of spontaneous abortion is unlikely to be a predictive factor for clinical UPD.

Most of the participants in our study cohort had UPD on a single chromosome. From our results, the distribution of UPD varied on different chromosomes; it was more likely to occur on chromosomes 16, X, and 2. However, Nakka et al. reported that UPD was most frequently observed on chromosomes 16, 4, 22, 1, and X [6]. This inconsistency may be attributed to the different study populations and the different identification approaches of UPD. In our institution, UPD test is recommended for cases with suspected sonographic presentation of genetic imprinting disorders, fetal abnormalities, or a history of previous adverse pregnancy. In the unfavorable outcome group, chromosomes 16 and 2 were the most frequently involved, and univariate logistic regression analysis showed that chromosomes 16 and 2 were risk factors for unfavorable outcomes.

Most UPD carriers have no clinical syndromes and are usually detected accidentally. However, if the UPD region involves an imprinted gene, the origin of UPD can lead to different clinical significances and outcomes [4, 10]. For example, when UPD occurs on chromosomes 6, 7, 11, 14, 15, and 20, it causes imprinted genetic disorders [15, 16]. Moore et al. investigated 35 newborns with severe FGR and found that maternal UPD on chromosome 16 was present in 5% of their cohort [17]. Kotzot, in 1999, reviewed abnormal phenotypes in UPD and found that FGR was reported in an increasing number of newborns with maternal UPD on chromosome 16 and also found that 4 out of 5 cases with maternal UPD on chromosome 2 were associated with FGR [18]. However, it remains difficult to prenatally establish the influence of UPD on all specific phenotypes. Our findings revealed that FGR was detected on chromosomes 16 and 2 in most patients with UPD. A previous study showed that FGR with postnatal growth failure is a common clinical phenotype associated with many UPDs and mainly occurs on chromosomes 2, 9, 16, and 20 [19, 20]. However, its disease-causing mechanisms are unclear and may be caused by imprinting disorders, recessive diseases, or confined placental mosaicism [21]. Among the six cases of FGR occurring on chromosome 16 reported by Xie et al., three cases expressed *CDT1* and two cases expressed *ALG1*. The database showed that the genes *CDT1* and *ALG1* were located at 16q24.3 and 16p13.3 and were associated with autosomal-recessive diseases associated with FGR [22]. Autosomal-recessive diseases caused by UPD are plausible explanations for FGR. In our cohort, three cases of fetuses with FGR were detected with a thickened and small placenta on prenatal ultrasound. We speculated that UPD on chromosome 16 correlated with FGR because UPD has a potential impact on placental function [11, 23]. It is known that FGR is a known risk factor for intrauterine demise, neonatal morbidity, and death.

Our study showed that approximately 61.8% of the UPD cases had ultrasound abnormalities, with multiple malformations being the most common. Fetuses with ultrasound abnormalities showed a higher rate of adverse pregnancy outcomes than those without ultrasound abnormalities, and the OR of unfavorable outcomes increased in fetuses with ultrasound abnormalities. Additionally, about half of the fetuses with UPD coexisted with FGR, all combined with other ultrasound abnormalities. A previous study demonstrated the similar result, with the data that the incidence of fetal or neonatal death was significantly higher in fetuses with absence of heterozygosity (AOH) and small for gestational age fetuses than the fetuses without [24]. Absence of heterozygosity could be classified as UPD or identity by descent (IBD), depending on its origin [19]. Prenatal ultrasound phenotypes of UPD can provide important information for predicting the prognosis.

The frequency of unfavorable outcomes of FGR was 85.7% in our study, which was higher than that in cases diagnosed with only UPD. The univariate regression analysis found that UPD cases with FGR had worse pregnancy outcomes than those without FGR. Therefore, focusing on fetal growth and development indicators can also predict adverse pregnancy outcomes from UPD.

### Clinical implications

This study demonstrated that about half of the fetuses with UPD coexisted with fetal growth retardation and the prognosis of fetuses with uniparental disomy combined with fetal abnormalities, especially fetal growth restriction, was much poorer than that of fetuses without fetal abnormalities. In clinical practice, combining sonographic presentations with the results of invasive prenatal diagnosis can provide useful information for clinical counseling.

### Research implications

The prenatal sonographic presentations differed greatly among different involved chromosome, with much poorer prognosis in fetuses with UPD 16 or UPD 2. The candidate genes for growth failure or genes with potential functional effects on placental insufficiency could be searched in UPD fetuses.

### Strengths and limitations

The strength of this study is that we focused on a confusing problem in genetic counseling. We report the rate of prenatal sonographic abnormalities and different chromosomes in UPD cases. Furthermore, we found that ultrasound abnormalities, fetal growth restriction, and uniparental disomy on chromosomes 16 or 2 are important in predicting uniparental disomy neonatal outcomes, which is very useful for clinicians and patients. Another strength is that SNP CMA was used on every case, enabling UPD to be detected.

Our study had several limitations. First, this was a retrospective single-center study. Second, the postnatal growth and development were not evaluated, and some patients were lost to follow-up, which may have led to an underestimation of the adverse phenotypes. Third, routine CMA may not detect complete or near-complete uniparental heterodisomy [11, 23], therefore, approximately 1/3 of UPD cases may not be detected. Fourth, definition of favorable outcome which was defined only as children being alive at the time of this writing. It did not provide the information of disability or development and children’s age at the time of the writing. Fifth, ultrasonographic findings could be operator dependent.

## Conclusions

We described the prenatal ultrasound features of UPD and analyzed the clinical pregnancy outcomes. Ultrasound abnormalities, FGR, and UPD on chromosomes 16 or 2 are important in predicting neonatal outcomes, particularly when fetal ultrasound abnormalities coexist with growth restriction. In clinical practice, sonographic presentations should be combined with the results of invasive prenatal diagnosis to provide useful information for clinical counseling.

### Electronic supplementary material

Below is the link to the electronic supplementary material.

Supplementary Material 1

## Data Availability

Data is provided within the manuscript. Please contact the primary corresponding author if someone wants to request the data from this study.

## Glossary

UPD: Uniparental disomy
SNP: Single-nucleotide polymorphism
CMA: Chromosome microarray analysis
CGH: Comparative genomic hybridization
NIPT: Noninvasive prenatal testing
CNVs: Copy number variations
FGR: Fetal growth restriction
IBD: Identity by descent
