# Supplementary material for: Prenatal ultrasound findings and clinical outcomes of uniparental disomy: a retrospective study
Source: BMC Pregnancy Childbirth. 2024 Apr 18;24:288. doi: 10.1186/s12884-024-06493-0 (PMC11027273; doi:10.1186/s12884-024-06493-0)
Supplement: Supplementary file 1 — Supplementary Material 1 [file 12884_2024_6493_MOESM1_ESM.docx]

| **Supplement Table 1: Ultrasound findings of 21 fetuses with UPD and combing with ultrasonographic abnormalities** | Pregnancy Outcomes | TOP 34 w | Selective reduction 32^+^w | TOP 29^+^w | TOP 34 w | TOP 24^+^w | TOP | TOP 33^+^w | | TOP 30 w | Selective reduction 22^+2^w | TOP | TOP 34^+4^w |
| --- | --- | --- | --- | --- | --- | --- | --- | --- | --- | --- | --- | --- | --- |
|  | Chromosome Microarray Analysis | 2p25,q37.3(15,702-242,775,901)×2hmz | Multiple UPDs in chromosome 1, 11,13, 14 | 16q21q22.3(60,136,253-73,345,707)×2hmz | 5q14.3q21.1(86,673,420-102,035,777)×2hmz | 2p25.3-p11.2(50,813-87,053,152)×2hmz  2q11.1-q37.3(95,550,957-242,773,583)×2hmz | Xp11.23p11.11(47,108,065-58,337,890)×2hmz | Multiple large fragment UPD in chromosome 2 | | 16q23.2q24.3(79,773,290-90,163,275)×2hmz | 16p13.3p12.3(7,717,327-19,208,505)×2hmz; 16q22.2q23.2(71,157,422-80,126,717)×2hmz | 15q14q25.3(36,248,978-88,131,599)×2hmz | 16q23.2q24.3(81,161,763-90,163,275)×2hmz |
|  | Karyotype | 46,XY | 46,XY | 46,XY | 46,XY | 46,XY | 46,XX | 46,XY | | 46,XX | 46,XX | 46,XX | 46,XX |
|  | Ultrasound Findings | FGR; short nasal bone; abnormal external geniatlia; thickened and  small placental | Absent right fibula | Ascites; peritonitis; thickened nuchal fold; intra-abdominal calcification;  cardiomegaly | Hypospadias | FGR; short long bone; hypospadias | Short long bone; thickened nuchal fold; single umbilical artery;  atresia of upper digestive tract | | FGR; oligohydramnios | FGR; absent nasal bone; descending aorta curving | FGR; cardiomegaly; intermittent disappearance of end diastolic blood flow of umbilical artery; descended RI of MCV | FGR; peritonitis; ascites; CPR＜1 | FGR; persistent left superior cavity |
|  | GW | 28^+2^ | 23^+3^ | 28 | 30^+3^ | 23^+3^ | 31^+5^ | | 29^+4^ | 24^+2^ | 21 | 22^+1^ | 22^+5^ |
|  | NO | 1 | 2 | 3 | 6 | 7 | 11 | | 12 | 13 | 14 | 18 | 22 |

| **Supplement Table 1 (supplement): Ultrasound findings of 21 fetuses with UPD and combing with ultrasonographic abnormalities** | Pregnancy Outcomes | TOP | TOP | Term birth, BW2.56kg | Preterm birth 30^+3^w, BW1.68kg | Preterm birth 33^+3^w, died 1 h after birth | TOP | Term birth, BW3.3kg, sever hydronephrosis | Term birth | Preterm birth 33^+3^w, BW1.45kg, respiratory distress syndrome | TOP | GW, gestational weeks; TOP, termination of pregnancy; BW, birth weight; FGR, fetal growth restriction; RI, resistance index; MCV, middle cerebral artery; CPR, cerebroplacental ratio; UPD, uniparental disomy; PSV, Peak systolic velocity. |
| --- | --- | --- | --- | --- | --- | --- | --- | --- | --- | --- | --- | --- |
|  | Chromosome Microarray Analysis | 17q12q22(33,848,718-53,405,927)×2hmz | 16q22.3q23.1(73738443-78629320)×2hmz 16q23.1q24.3(78700673-90163275)×2hmz | 15q25.1q26.1(79,396,088-87,631,279)×2hmz | 1q43q44(238,466,998-249,198,692)×2hmz | Multiple large fragment UPD in chromosome 2 | 16p13.3(89560-7064036)×2hmz | 8q12.1q13.3(57,492,825-72,184,919)×2hmz | Multiple large fragment UPD in chromosome 16 | Paternal UPD X | 14q32.2q32.33(97170185-107285437)×2hmz |  |
|  | Karyotype | 46,XX | 46,XX | 46,XX | 46,XX | 46,XX | 46,XX | 46,XY,qh+ | 46,XX | 46,XX | unknown |  |
|  | Ultrasound Findings | FGR; femoral fracture | FGR; thickened and abnormal echo of placental | Choroid plexus cyst; double renal pelvis | Ascites; enhanced intestinal echo; intestinal dilation | FGR; short nasal bone; cerebral ventriculomegaly; persistent right umbilical vein | FGR; CPR＜1; thickened and small placental | Bilateral hydronephrosis | FGR; cardiomegaly | FGR; abnormal echo of placental (partal hydatidiform sign); increased MCA-PSV | FGR; enlarged cisterna magna |  |
|  | GW | 29 | 16 | 21^+4^ | 29^+3^ | 29^+2^ | 25^+5^ | 31^+5^ | 27 | 17 | 22^+6^ |  |
|  | NO | 24 | 25 | 26 | 27 | 28 | 29 | 30 | 32 | 33 | 34 |  |
